# Sperm Competition in Humans: Mate Guarding Behavior Negatively Correlates with Ejaculate Quality

**DOI:** 10.1371/journal.pone.0108099

**Published:** 2014-09-24

**Authors:** Samantha Leivers, Gillian Rhodes, Leigh W. Simmons

**Affiliations:** 1 Centre for Evolutionary Biology & School of Animal Biology, University of Western Australia, Crawley, Australia; 2 ARC Centre of Excellence in Cognition and its Disorders, School of Psychology, University of Western Australia, Crawley, Australia; Brock University, Canada

## Abstract

In species where females mate with multiple males, the sperm from these males must compete to fertilise available ova. Sexual selection from sperm competition is expected to favor opposing adaptations in males that function either in the avoidance of sperm competition (by guarding females from rival males) or in the engagement in sperm competition (by increased expenditure on the ejaculate). The extent to which males may adjust the relative use of these opposing tactics has been relatively neglected. Where males can successfully avoid sperm competition from rivals, one might expect a decrease in their expenditure on tactics for the engagement in sperm competition and vice versa. In this study, we examine the relationship between mate guarding and ejaculate quality using humans as an empirical model. We found that men who performed fewer mate guarding behaviors produced higher quality ejaculates, having a greater concentration of sperm, a higher percentage of motile sperm and sperm that swam faster and less erratically. These effects were found independent of lifestyle factors or factors related to male quality. Our findings suggest that male expenditure on mate guarding and on the ejaculate may represent alternative routes to paternity assurance in humans.

## Introduction

In species where females mate with multiple males, the sperm of two or more males must compete to fertilise available ova [1]. Selection from sperm competition is expected to favor opposing adaptations that function either in the avoidance of or engagement in sperm competition [2], [3]. Adaptations for the avoidance of sperm competition can include the use of mate guarding or anti-aphrodisiac odors and copulatory plugs, while adaptations for the engagement in sperm competition include copulation frequency and duration or strategic adjustments in ejaculate quality [4]–[6].

Investment in behavioral mate guarding is likely to represent a significant cost for males as it reduces their ability to perform other ecologically important behaviors such as territorial patrol, foraging and pursuing additional mating partners [7]–[10]. Therefore, males should only invest in mate guarding when the reproductive benefits of mate guarding outweigh the costs. Indeed, there is evidence to show that males will adjust their investment in mate guarding dependent on the perceived risk of sperm competition from rival males [2], [11], [12]. Likewise, physiological investments into ejaculate production are costly for males, and there is widespread evidence that males will also adjust their investment into the ejaculate dependent on their perceptions of sperm competition risk [for a review, see 4]. However, little is known about how individual males might balance their investments into tactics for the engagement in and avoidance of sperm competition when paired with a given female. Where mate guarding is highly effective we might expect males to reduce their investment into physiologically expensive ejaculates. Conversely, were males unable to effectively guard their mates, we might expect them to increase their expenditure on the ejaculate.

Some evidence is available to suggest that male expenditure on the engagement in sperm competition may be negatively associated with their expenditure on its avoidance. For example, in a number of colonial bird species, at least one member of the breeding pair is required to protect the nest site from intruders, resulting in periods of time in which a male is unable to guard his mate [13]. Therefore, preventing the female from engaging in extra-pair copulations through mate guarding can be compromised, so that males may increase the use of tactics for the engagement in sperm competition. Indeed, in a comparative study of 173 bird species, Møller and Birkhead [14] found that species in which males were limited in their use of mate guarding had an increased frequency of in-pair copulations (IPCs). As the outcome of sperm competition is often influenced by the quantity of a male's sperm in the female's reproductive tract [15]–[18], the increased use of IPCs likely functions in competing for paternity when males are unable to avoid sperm competition through mate guarding.

Further evidence can be found within species where males adopt alternative mating tactics [19]. For example, in the Mediterranean wrasse [20] there are three distinct male phenotypes that differ in their reproductive strategies for achieving paternity success. Satellite and nesting males actively guard their mates from rival males whilst sneaker males ‘sneak’ copulations and therefore gain reproductive success through sperm competition alone. Sneaker males produce ejaculates of higher quality than both satellite and nesting males, suggesting that male investment in tactics for the engagement in sperm competition and its avoidance are dependent upon the mating strategy adopted. Whether a negative association between tactics for the avoidance of and engagement in sperm competition is present within species that lack discreet alternative reproductive tactics has not been studied.

Here, we examine the association between expenditure on mate guarding and the ejaculate using humans as an empirical model. Human sperm competition is a hotly debated topic [for a review, see 21]. Humans are generally considered socially monogamous, with limited evidence for polygyny [22], [23], suggesting that humans are subject to relatively weak selection from sperm competition. Indeed, humans lack morphological indicators of sperm competition, such as large relative testes size [24], [25]. However, female extra-pair copulations in humans are relatively common with approximately 20% of women reporting cases of sexual infidelities [24], [26] and there is considerable evidence that men have evolved behavioral adaptations that function in the prevention of extra-pair copulations [27]–[29]. For example, mate guarding has been well studied in humans and has been documented across cultures as a tactic that functions to avoid sperm competition by preventing females from engaging in copulations with rival males [27], [28], [30]–[32]. Men may also have developed behavioral and physiological tactics for the engagement in sperm competition. Men show an increased interest in IPCs as the period of time between the couple's last copulation increases [33] and there is also evidence to suggest that men increase their investment in ejaculate quality when responding to explicit images depicting sperm competition [34].

Considering our hunter-gatherer origins, it seems likely that men would have been limited in their ability to continuously guard their mates from rival males due to ecological constraints. Anthropological data suggest that extra-pair sex was prevalent across many preindustrial societies [35], [36] so that humans, like other animals, are expected to have evolved mechanisms for the engagement in and avoidance of sperm competition [21]. When the relationship between mate guarding and IPC frequency was explored in humans, it was found that those men who performed a high number of mate guarding behaviors *also* engaged in more frequent IPCs [37]. These findings might suggest a positive relationship between tactics for the engagement in and avoidance of sperm competition in humans. However, IPC frequency may be a poor indicator of a man's investment in tactics for the engagement in sperm competition as women can also initiate copulations [38]. Furthermore, mate guarding and copulation are not necessarily mutually exclusive behaviors; the more time a man spends with his mate, the greater opportunity he has to pursue copulations with her. Indeed, IPC frequency could arguably be considered a form of mate guarding, potentially reducing a females tendency to seek extra-pair copulations, which would account for the positive relationship between mate guarding behavior and IPC frequency. A physiological trait important in the engagement in sperm competition that has been widely researched in non-human animals, but rarely in humans, is ejaculate quality [4]. Here we examined the relationship between mate guarding and ejaculate quality in humans and hypothesize that men will show a negative correlation between their use of mate guarding behaviors and ejaculate quality because men who spend more effort guarding are expected to face a lower risk of sperm competition.

Forty-five male participants in committed heterosexual relationships were asked to provide a semen sample and information about their use of mate guarding behaviors. Research on both human and non-human animals has suggested that male quality can be associated with both ejaculate quality and mate guarding behavior [4], [20], [39]–[42]. We therefore determined whether male mate value might account for variation in ejaculate quality and mate guarding, by collected information on three measures of male quality — self-perceived mate value, and female perceived dominance and attractiveness.

## Materials and Methods

### Participants

Forty-five male participants who were in committed heterosexual relationships were recruited from the University of Western Australia community and other universities in the Perth metro area. All participants were of Western European descent in order to control for the possible influence of race on ejaculate parameters [43], [44]. Participants were aged between 18 and 35 years (mean ± 1SD, 24.20±4.72), as sperm quality is known to decline after the age of 35 years [45].

### Ethics statement

Ethics approval for this research was granted by the UWA Human Ethics Research Committee (project number RA/4/1/5012). Participants read an information sheet detailing their role in the study and provided written consent prior to commencing the study. Participants nominated a unique 4-digit participant code, which was written on all materials in order to allow the cross-referencing of data, and to ensure anonymity.

### Procedure

#### i) Laboratory visit

Participants were first asked to read an information sheet and sign a consent form. Participants then completed a Lifestyle Survey in order to take into account the influence of environmental factors on sperm quality [34]. For example, participants reported their frequency of sexual activity during an average week so that this could be controlled statistically in our analyses. After the Lifestyle Survey, men completed the Mate Retention Inventory-Short Form designed to measure their use of mate guarding behaviors [MRI-SF, 46]. The MRI-SF is a shortened form of the original Mate Retention Inventory (MRI) that assesses the frequency with which an individual performs a number of mate guarding behaviors [27], [47]. The MRI-SF assesses the performance of 38 behaviors, and responses from the MRI-SF have been shown to have good validity, strong internal consistency and a positive relationship with responses collected from the original 104-item MRI [46]. MRI-SF scores (hereafter ‘mate guarding’) were calculated by summing responses so that high scores indicated high use of mate guarding behaviors.

To obtain measures of self-perceived mate value, participants completed the Components of Self-Perceived Mate Value questionnaire (CSMV). The CSMV combines and evaluates a number of pre-existing measures of self-perceived male mate value and produces distinct factors that account for variance in self-perceived male mate value [48]. Items from the CSMV questionnaire were summed to produce an overall CSMV score (hereafter ‘mate value’, [48], [49], [50]). The items “I often stay at home because I have nothing to do”, “I would like members of the opposite sex to hit on me more than they do” and “I often worry about not having a date” were reverse loaded so that high scores indicated a high self-perceived mate value.

Full-body photographs of each participant were taken and rated at a later date for attractiveness or dominance by 30 heterosexual women of Western European descent (15 rated attractiveness, 15 rated dominance) on a scale of 1 (“Not at all attractive/dominant”) to 7 (“Very attractive/dominant”). Attractiveness is well known as a component of mate value [51], [52] and is associated with male mating success [53], [54]. Dominance is also an important factor that contributes to male mate value [55], [56]. Rated dominance from images has been linked with real-life dominance in the workplace [57] and mating success [56]. Participants wore shorts and a t-shirt for the photograph and were told to assume a neutral expression. Photographs were scaled to a portrait orientation of 1232 × 816 pixels and shown at a resolution of 1608 × 1050 pixels on a 20” screen iMac. Images were viewed at an approximate distance of 60cm, at a vertical visual angle of approximately 16.4 degrees and a horizontal visual angle of approximately 4.8 degrees. Photoshop was used to remove jewelry and tattoos, color clothing black and to color the background white.

Participants were given an envelope containing a 70ml container for sample collection, aluminum foil and an Ejaculate Information Sheet, which was used to collect information on the time the sample was collected, the proportion of the sample collected and the time taken to collect the sample. We also asked how long it had been since the participant's previous ejaculation. This allowed us to take into account the participant's most recent sexual activity and thus control statistically for the possible effects of sperm depletion in our analyses. Participants were given verbal instructions outlining the collection process at this time and had the opportunity to ask any questions.

#### ii) Semen collection

Participants collected their semen sample in their own home via masturbation. Subjects were instructed not to use any erotic stimuli during collection and were asked to abstain from all sexual activity for at least 48 hours, but no longer than 6 days before collection. Samples were produced after 07:00 and deposited in the container provided. After collection, the participants were instructed to wrap the container in the provided foil and store in a warm place whilst in transit to the laboratory. Participants then completed the Ejaculate Information Sheet. The sample was returned to the laboratory with all other materials within 60 minutes of collection and by no later than 09:30. Upon delivery, participants received a debrief information sheet and remuneration of $20AUD.

### Semen analysis

Semen analysis was conducted using Hamilton Thorne Computer Assisted Semen Analysis (CASA) immediately after delivery, following the protocols outlined by the World Health Organisation [58]. CASA technology is used in both clinical and experimental settings and there is evidence that sperm quality parameters assessed via CASA predict fertilization success in both human and in non-human species (e.g. [59], [60]–[62]). Aliquots of 2microliters of the semen sample were pipetted into each of the chambers of a pre-warmed Leja Standard Count 4 chamber slide which was placed onto a pre-warmed Hamilton Thorne HTM MiniTherm stage warmer set to 37 Celsius. Data were collected on sperm concentration, percentage of motile sperm, average path velocity (VAP), straight line velocity (VSL), velocity along the sperm cells point-to-point track (VCL), the lateral amplitude of sperm head movement (ALH), the frequency with which the sperm head crosses the average sperm path (BCF), the straightness of the sperm's path (STR), and the linearity of the sperm's path (LIN). One scan was taken from each of the four chambers followed by a further two scans from two chambers chosen at random, which were then averaged to produce mean values for the sperm parameters.

If the CASA was unable to analyze a sample due to a very high sperm concentration (*N* =  4), a proportion of the semen sample was centrifuged for six minutes at 13000RPM in an Eppendorf Centrifuge 5804 to separate the sperm and seminal plasma. The seminal plasma was then used to dilute a known volume of the original sample. A normal analysis was then carried out and the correct sperm concentration was calculated after analysis.

After analysis, all contaminated materials were sanitized in a 1:10 mix of household bleach and water before being disposed of in a laboratory bin.

## Results

### Semen quality

Four subjects were excluded from analysis (leaving 41 participants) because their semen samples had concentration values of less than 15 million sperm per ml, which is below the lower reference limit for a normal sample according to the World Health Organisation [58]. Concentration values were log transformed to achieve normality. As semen parameters were highly correlated (see Table S1), we followed the protocol employed by Agarwal, Sharma and Nelson [63] whereby all sperm quality parameters were entered into a principal components analysis to produce principal components that accounted for variance in ejaculate quality. Three principal components with eigenvalues greater than 1 were extracted and were found to account for 88.48% of the variance in ejaculate quality (Table 1).

**Table 1:** Principal components analysis on all sperm quality parameters to produce principal components that account for sperm quality.

	PC1	PC2	PC3
**VAP**	.905	.373	.125
**VSL**	.959	.146	.182
**VCL**	.516	.805	.274
**ALH**	−.178	.928	.203
**BCF**	.054	−.194	.820
**STR**	.597	−.647	.340
**LIN**	.700	−.701	−.003
**% motile**	.773	.146	−.353
**concentration**	.532	.078	−.689
**% variance in sperm quality explained**	41.85%	29.35%	17.26%

VAP =  average path velocity, VSL =  straight line velocity, VCL =  velocity along the sperm cells point-to-point track, ALH =  lateral amplitude of sperm head movement, BCF =  frequency with which the sperm head crosses the average sperm path, STR =  straightness of the sperm's path, LIN =  linearity of the sperm's path, % motile =  percentage of motile sperm in the ejaculate, concentration =  concentration of sperm in the ejaculate (million sperm/ml).

PC1 described ejaculates with high motility and high swimming speeds, being most heavily loaded by the percentage of motile sperm in the ejaculate, VAP, VSL and LIN, which have all been linked to fertilization success in IVF treatments [60], [64]. PC2 described the curvature of the sperm path, being positively loaded by VCL and ALH and negatively loaded by LIN. PC3 was positively loaded by BCF and negatively loaded by concentration and described samples characterized by low concentration with erratically moving sperm. PC3 was reversed scored so that high values indicated high concentration and less erratic sperm in order to allow direct comparisons with PC1 and PC2.

All three sperm quality variables were run independently in General Linear Models (GLM) with all lifestyle and collection variables from the Lifestyle Survey and Ejaculate Questionnaire included. Non-significant terms were removed from the model in a stepwise procedure [65]. PC1 was influenced by the period of time between the semen sample's collection and its analysis (*F*_1,29_ =  10.96, *P* =  0.002), by how much alcohol the participant consumed in an average week (*F*_2,29_ =  5.63, *P* =  0.009), and whether or not the participant consumed caffeinated beverages (*F*_1,29_ =  11.78, *P* =  0.002). PC2 was influenced by the length of time between the participant's experimental sample and their last ejaculation (*F*_1,31_ =  7.14, *P* =  0.012) and by the participant's frequency of sexual activity in an average week (*F*_1,31_ =  20.46, *P*<0.001). PC3 was influenced by the length of time between the participant's experimental sample and their last ejaculation (*F*_1,32_ =  4.75, *P* =  0.037). All contributing lifestyle and collection variables were controlled for in subsequent analyses.

### Mate guarding and mate value

In all subsequent analysis, a subset of 34 participants were analyzed as seven participants had missing or incomplete independent variables that necessitated exclusion. Female raters showed good consensus on their ratings of attractiveness (2.9±0.9, Cronbach's alpha = .97) and dominance (4.0±1.1, Cronbach’s alpha = .89). Self-perceived mate value did not correlate with either attractiveness (*r*_34_ =  −0.13, *P* =  0.468) or dominance scores (*r*_34_ =  0.03, *P* =  0.853), but attractiveness and dominance were positively correlated (*r*_34_ =  0.45, *P*<0.001). We found no evidence of a relationship between mate guarding and male quality, with mate guarding showing no significant correlations with self-perceived mate value (*r*_34_ =  0.13, *P* =  0.481), attractiveness (*r*_34_ =  −0.16, *P* =  0.376) or dominance (*r*_34_ =  −0.28, *P* =  0.119).

### Relationship between mate guarding and ejaculate expenditure

**Figure 1:**
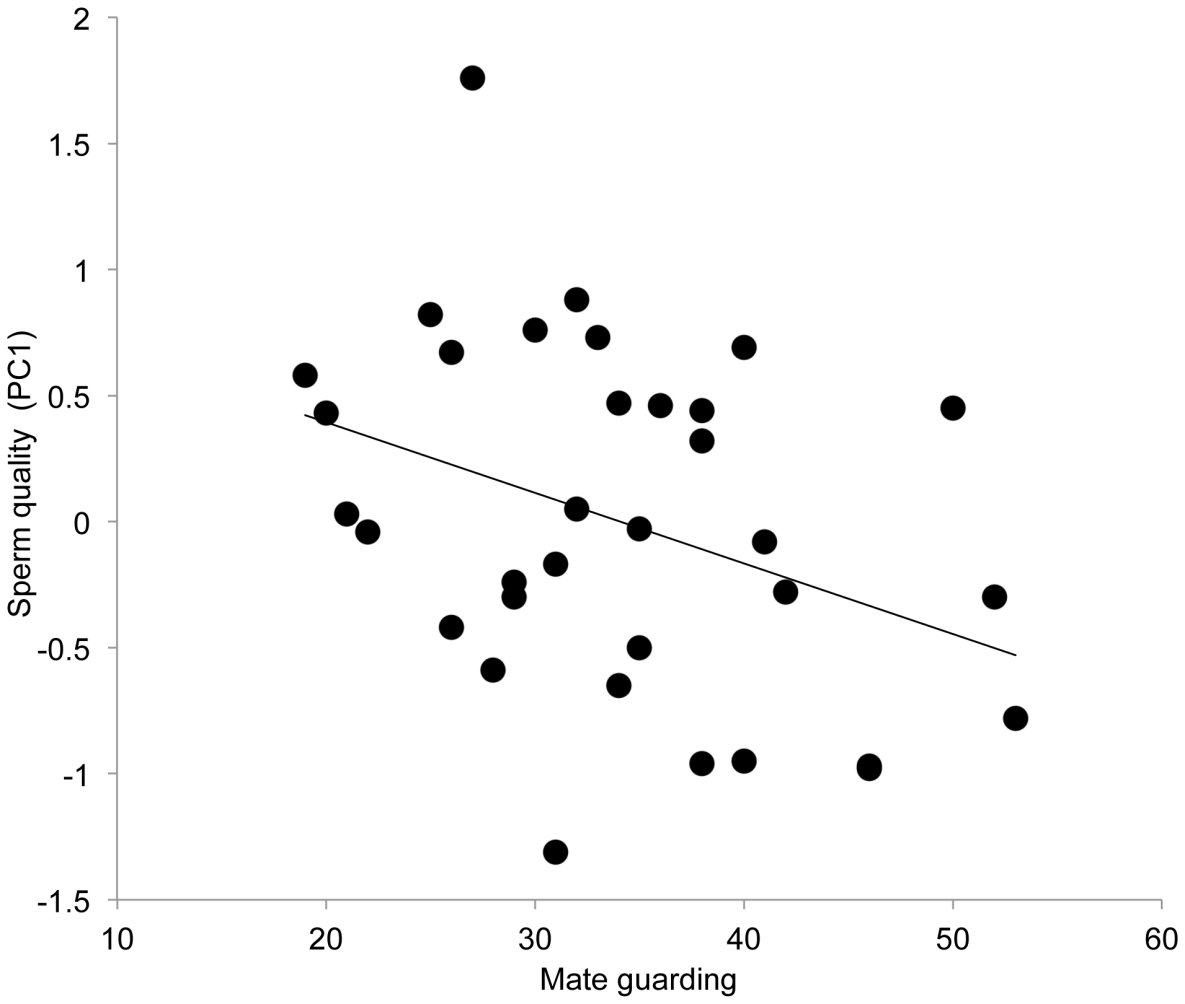
Relationship between mate guarding frequency and PC1 (after accounting for the influence of lifestyle and collection variables), which describes ejaculates with a high percentage of motile sperm and high swimming speed.

PC1 was entered as the dependent variable into GLMs with all three male mate value variables (attractiveness, dominance, self-perceived mate value) and mate guarding entered as predictor variables. A significant main effect of mate guarding was found (*F*_1,28_ =  4.51, *P* =  0.043), with a negative relationship between the performance of mate guarding behaviors and PC1 (effect size: *β* =  −.030, 95% CI [−.059, −.001], *R^2^* change = .072, Fig. 1), indicating that participants who invested less in mate guarding behaviors produced ejaculates within which the motility of sperm was greater. None of the male mate value variables accounted for any variance in PC1 and were dropped from the final model.

**Figure 2:**
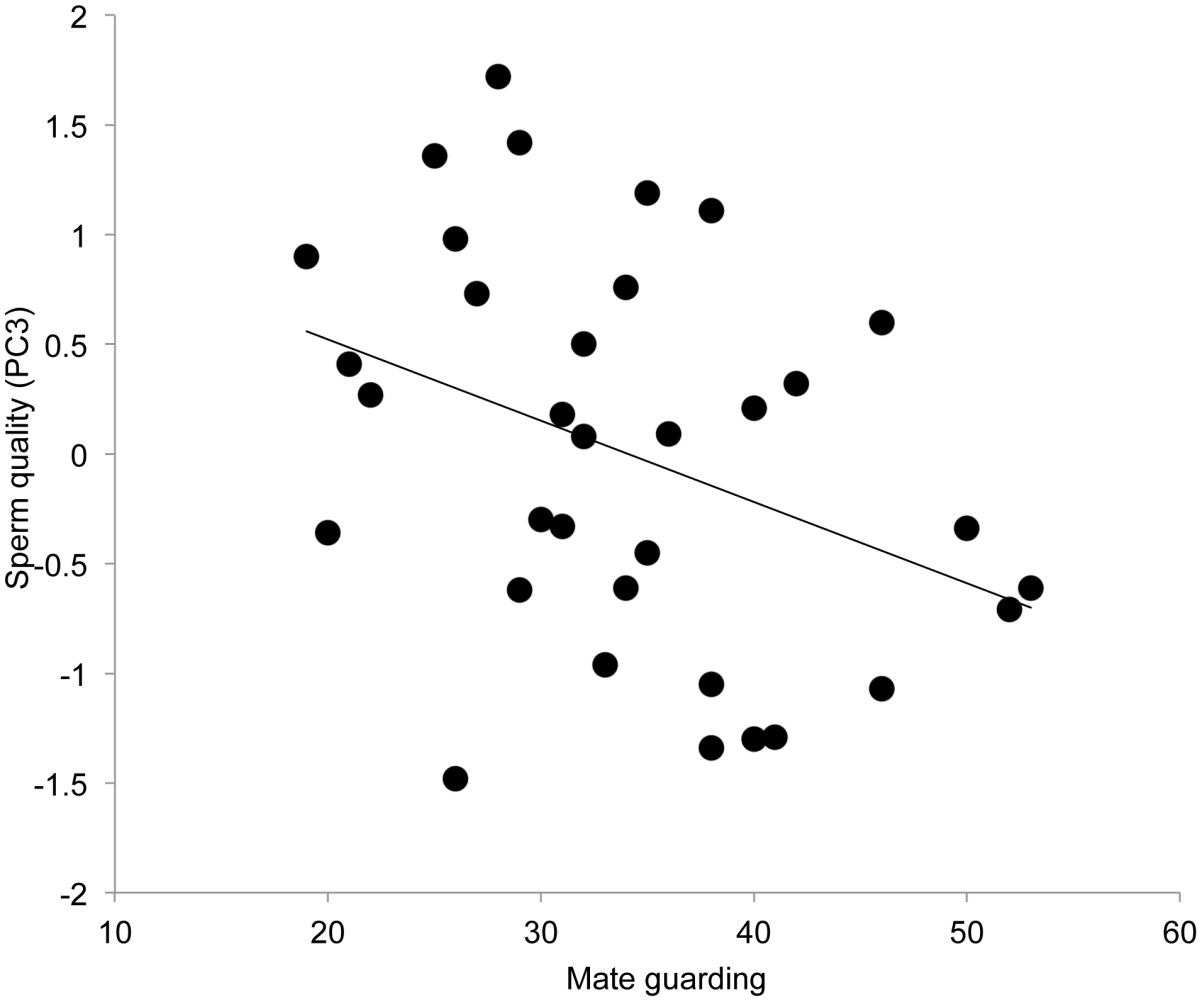
Relationship between mate guarding frequency and the unstandardized residuals of PC3 (after accounting for the influence of lifestyle and collection variables), which describes ejaculates with a high concentration of sperm and sperm that do not move erratically.

The same GLM was run for both ejaculate quality PC2 and PC3. There were no significant main effects of variables of interest on PC2, but PC3 showed a significant main effect of mate guarding (*F*_1,31_ =  4.887, *P* =  0.035) with a negative relationship between the performance of mate guarding behaviors and PC3 (effect size: *β* =  −.037, 95% CI [−.071, −.003], *R^2^* change = .119, Fig. 2), indicating that participants who invested more in mate guarding behaviors had lower numbers of sperm in their ejaculate and sperm that moved more erratically. Again, male mate value variables were not significant and were dropped from the final model.

According to guidelines from Field [66], PC1 and PC2 were acceptable for analysis as both PCs had high factor loadings with at least four loadings of 0.6 or greater. However, PC3 had only two factor loadings greater than 0.6: concentration and BCF. For this reason, to confirm the significance of the observed patterns of variation we also analyzed concentration independently. Concentration is the strongest predictor of fertility in men [61] and is the most widely used indicator of ejaculate quality in sperm competition research [4]. Concentration was not influenced by any lifestyle or collection variables and showed a significant main effect of mate guarding (*F*_1,32_ =  5.087, *P* =  0.031, effect size: *β* =  −.014, 95% CI [−.026, −.001], *R* ^2^ = .137), indicating that participants who invested more in mate guarding behaviors had lower numbers of sperm in their ejaculate. This analysis corroborates our analyses based on principal components.

## Discussion

Our study tested the general hypothesis that among males there would be a negative relationship between the use of tactics for the engagement in and avoidance of sperm competition, by examining the relationship between mate guarding and ejaculate quality in humans. Our results provide support for this hypothesis. Men who performed more mate guarding behaviors produced lower quality ejaculates, having a lower concentration of sperm, a lower percentage of motile sperm and sperm that swam slowly and erratically. These effects were independent of lifestyle or collection variables, and were not predicted by men's self-perceived mate value, attractiveness or dominance. Given the personal nature of the task required, recruitment of subjects for studies such as ours is difficult, and our study was limited in its sample size so that the 95%CIs on our observed effect sizes were broad. Nonetheless, the relationship was confirmed using two independent sperm quality indices, which together accounted for 59.1% of the variance in ejaculate quality. Previous research has found evidence for a negative relationship between male expenditure on tactics for the engagement in and avoidance of sperm competition, both across species [14], and within species with discrete alternative mating strategies [20]. Our results provide evidence for a continuous negative relationship between these opposing sperm competition tactics in a species without discrete alternative tactics [24], [67].

Shackelford, Goetz, Guta and Schmitt [37] found a positive relationship between the use of mate guarding and IPCs in humans. However, their use of IPC frequency as a measure of male engagement in sperm competition assumes that men control the rate of IPCs, which is not necessarily the case. Females will play a significant role in sperm competition and its avoidance [68], and women will initiate IPCs, particularly when at the most fertile point of their cycle [38]. The fact that women can initiate IPCs makes IPC frequency a relatively poor measure of male expenditure on sperm competition compared with ejaculate quality. Indeed, women may be selected to initiate more IPCs with males who show high use of mate guarding behaviors in order to ensure fertility, precisely because such males invest less in their ejaculate. For example, work on fishes has shown that male expenditure on mate guarding comes at a cost of reduced expenditure on the ejaculate with the consequence that female fertility is reduced [69].

Unlike IPC frequency, ejaculate quality is unlikely to be directly influenced by the female and certainly not in the context of our experimental design. For example, one possible reason for the negative relationship between mate guarding behavior and ejaculate quality could be sperm depletion. If frequent IPC is a behavior used by males as part of their mate guarding, or if females initiate more IPCs with mate guarding males, then those males who guard more strongly would be expected to have lower semen quality because of their greater mating frequency. However, we controlled statistically for variation in ejaculate quality that was due to the total amount of sexual activity men reported per week, and importantly the time since their last ejaculation. Therefore, the pattern of correlation we observe is independent of any influence of IPC frequency or sperm depletion.

The negative relationship between mate guarding and sperm quality obtained here could potentially be explained by their mutual covariation with male mate value. High quality men (with better quality ejaculates) may invest less in mate guarding because their partners are less likely to seek extra-pair copulations. Conversely, men of low mate value (with poorer quality ejaculates) may invest more in mate guarding because they are at a greater risk of having their mate defect from the relationship. Our mate value parameters included independently female assessed measures of mate quality (attractiveness and dominance) as well as a measure of men's self-perceived mate value. These measures are likely to capture much of the variance in male mate value, yet none of them were associated with ejaculate quality or mate guarding behavior. The negative association between ejaculate quality and mate guarding behavior we have observed is thus unlikely to be mediated by their mutual covariation with mate value.

The correlation between mate guarding and ejaculate quality we have observed could arise because of fixed genetic differences among men in their expenditure on these traits and/or through socially cued phenotypic plasticity. Cross cultural studies have shown how both men and women's sociosexuality — their tendency to engage in behaviors that generate sperm competition — are linked to personality types [70] that exhibit some degree of underlying genetic variation [71] and twin studies have found significant additive genetic variation for sociosexuality itself [72]–[74]. Likewise, ejaculate quality has been shown to exhibit significant additive genetic variance [75] Thus, on the one hand it is possible that men may have fixed, genetically determined expenditures on mate guarding and ejaculate quality, and future work should establish the extent to which mate guarding exhibits genetic versus environmental variation and the extent to which mate guarding and ejaculate quality are genetically correlated. On the other hand, men can also show phenotypic plasticity in ejaculate quality. There is considerable research to show that ejaculate quality can be context dependent [76], [77]. For example, Kilgallon and Simmons [34] found that both among and within subjects, when men viewed images depicting sperm competition scenarios they produced ejaculates containing sperm of greater motility than when they viewed images of women alone. Thus, environmental constraints on a male's ability to mate guard might generate plasticity in ejaculate quality. For example, males might respond to direct feedback from their mate; if a man's partner were to actively avoid being mate guarded, he may invest more in his ejaculate. Further research examining socially cued plasticity in both mate guarding and ejaculate quality is warranted.

In conclusion, our findings suggest that male expenditure on mate guarding and on the ejaculate can represent alternative means by which males respond to sperm competition. Men who performed fewer mate guarding behaviors to avoid sperm competition had higher quality ejaculates for the engagement in sperm competition. This relationship between mate guarding and ejaculate quality was independent of male quality. Future research is needed, to replicate our findings, to determine the extent to which this correlated expenditure occurs across other taxa, and to identify the genetic, and environmental and ecological factors that influence the relationship between the use of tactics for the engagement in and avoidance of sperm competition.

## Supporting Information

Table S1 **Correlation matrix between all sperm parameters (Pearson's correlations are shown above the diagonal and Spearman's correlations are included below the diagonal for comparison).**
(DOCX)
