# Supplementary material for: Sperm Competition in Humans: Mate Guarding Behavior Negatively Correlates with Ejaculate Quality
Source: PLoS One. 2014 Sep 24;9(9):e108099. doi: 10.1371/journal.pone.0108099 (PMC4176016; doi:10.1371/journal.pone.0108099)
Supplement: Table S1 — Correlation matrix between all sperm parameters (Pearson's correlations are shown above the diagonal and Spearman's correlations are included below the diagonal for comparison). (DOCX) [file pone.0108099.s001.docx]

|  | **Concentration** | **% motile** | **VAP** | **VSL** | **VCL** | **ALH** | **BCF** | **STR** | **LIN** |
| --- | --- | --- | --- | --- | --- | --- | --- | --- | --- |
| **Concentration**  **% motile**  **VAP**  **VSL**  **VCL**  **ALH**  **BCF**  **STR**  **LIN** | .594**  .372*  .338  .150  -.088  -.450**  -.052  .276 | .591**  .620**  .549**  .370*  .010  -.471**  .040  .378* | .398*  .619**  .961**  .814**  .277  -.027  .184  .354* | .371*  .578**  .972**  .677**  .104  .004  .426*  .538** | .187  .420*  .861**  .749**  .729**  .033  -.231  -.174 | -.041  .010  .281  .111  .702**  -.010  -.570**  -.682** | -.444**  -.476**  -.121  -.079  -.054  -.045  .070  -.157 | -.039  .075  .244  .460**  -.140  -.579**  .132  .861** | .237  .323  .344*  .530**  -.156  -.737**  -.029  .883** |

**Table S1** Correlation matrix between all sperm parameters (Pearson’s correlations are shown above the diagonal and Spearman’s correlations are included below the diagonal for comparison).

VAP= average path velocity, VSL= straight line velocity, VCL= velocity along the sperm cells point-to-point track, ALH= lateral amplitude of sperm head movement, BCF= frequency with which the sperm head crosses the average sperm path, STR= straightness of the sperm's path, LIN= linearity of the sperm's path, % motile= percentage of motile sperm in the ejaculate, concentration= concentration of sperm in the ejaculate (million sperm/ml).

* P< 0.05

**P< 0.01
